# A mixed methods study of wellbeing and resilience of undergraduate nursing students: implications for the post-pandemic era

**DOI:** 10.1186/s12912-025-03066-0

**Published:** 2025-04-11

**Authors:** Kim Foster, Louise Alexander, Michael Steele, Tyneille Hale, Larissa Hutchison, Ronald Trejo, Johanna Boardman

**Affiliations:** 1 School of Nursing, Midwifery and Paramedicine, Australian Catholic University, Level 5, 215 Spring Street, Melbourne, VIC 3000 Australia; 2 School of Nursing & Midwifery, Deakin University, Burwood, Australia; 3 Institute for Health Transformation, Deakin University, Burwood, Australia; 4 School of Allied Health, Australian Catholic University, Brisbane, Australia

**Keywords:** Post-pandemic era, Coping, Mixed methods, Undergraduate nursing students, Resilience, Wellbeing

## Abstract

**Background:**

The COVID-19 pandemic and rapid shift to online learning have had ongoing impacts on nursing students’ wellbeing and resilience. We are yet to fully understand the implications for this emerging workforce in the post-pandemic era. The aims of this mixed methods study were to investigate wellbeing, coping and resilience of undergraduate nursing students in the pandemic; explore relationships between these variables and investigate predictors of wellbeing and coping, including differences between domestic and international students; explore how students experienced and managed adversity; understand how their mental distress and wellbeing were influenced by resilience resources used to deal with adversity, and identify implications for nurse wellbeing as they enter the workforce in the post-pandemic era.

**Methods:**

A convergent mixed methods design was used. An online survey investigated wellbeing (COMPAS-W), psychological distress (DASS-21), coping (Brief COPE) and resilience (ARM-R) was completed by *n* = 175 undergraduate nursing students. Semi-structured interviews with *n* = 18 students explored how they navigated challenges. Descriptive, correlational, and regression analyses, and thematic analysis, were conducted. Mixed methods analysis was used to integrate both sets of findings.

**Results:**

Students reported high levels of mental distress, yet also moderate levels of wellbeing and resilience. Key findings included domestic students reporting significantly greater stress than international students, and wellbeing being predicted by lower mental distress and increased problem-focused coping. Students coped with challenges by being proactive, drawing on a range of coping strategies, and seeking technical and emotional support. From a social-ecological resilience perspective, access to and engagement with a range of personal, environmental and relational resources served as protective factors for their wellbeing.

**Conclusions:**

This study provides valuable new insights into protective factors for nurses during a period of extraordinary challenge. In the post-pandemic era, there is a need to strengthen the wellbeing and retention of new graduates now entering the workforce from university. Implementation of targeted strategies to strengthen graduates’ peer relationships and sense of belonging at work, and wellbeing and resilience education, are needed. Longitudinal follow-up of graduates’ wellbeing is recommended.

**Supplementary Information:**

The online version contains supplementary material available at 10.1186/s12912-025-03066-0.

## Background

The global public health crisis of the COVID-19 pandemic required sudden and substantial changes to the delivery of higher education, where universities needed to rapidly move courses online. Transition to online learning occurred so quickly that staff and students were largely unprepared for such a significant shift [1], which led to challenges for student learning, including student engagement, digital literacy, and self-motivated learning [2].

For undergraduate/pre-registration nursing students, evidence indicates the rapid shift to online learning during the pandemic had both positive and negative impacts on their learning. Students enjoyed the flexibility of online approaches [3] which included engaging with learning material on their own schedule [4] and revisiting learning content to ensure sufficient comprehension [5]. However, students also reported substantial challenges, including concerns about academic progress, low satisfaction with the learning experience, and social isolation [3]. Nursing students experienced anxiety related to reliable internet access [1], computer skills illiteracy [6], and clinical placement preparedness and practical skills competency [4]. Students also had difficulties with concentration and self-regulation of learning [7]. Therefore, while students appreciated the flexibility of online learning, they had challenges with self-directed learning, concerns regarding clinical skills acquisition, and a lack of social connectedness with peers.

The pandemic also impacted nursing students’ levels of mental distress, and their wellbeing. They not only had to grapple with the sudden shift to online learning and impacts on knowledge and clinical skill development, but also with the effects the pandemic had on their mental health. A review found one-third of nursing students experienced anxiety and stress, and one-half reported depression during the pandemic [8]. A further study of Australian nursing students reported even higher levels, with mental health problems experienced by over half the sample, and 65% seeking professional help and/or treatment, and final year students reporting more distress than other students [9]. Students’ mental distress is likely to have an ongoing impact on their mental health and wellbeing in the post-pandemic period [10]. Previous findings indicate the need to better understand students’ use of coping strategies and resilience resources in the context of the pandemic [11].

Coping is how people manage stressors and is critical to overcoming stressful challenges [12]. Adaptive coping strategies help people problem solve or regulate emotions around a stressor [13], whereas maladaptive coping typically engages strategies which avoid or distract from the stressor, and is associated with negative outcomes, including hindered learning and poor mental health [14]. In the context of the pandemic, nursing students were found to engage in a range of coping strategies [15, 16]. Adaptive strategies included information seeking, humour, relaxation, optimism and family support [16]. Maladaptive strategies included behavioural disengagement, self-blame and substance use [15]. Some nursing students reported experiencing overwhelm and were consequently *not* coping [17]. This indicates variability in nursing students’ responses to the COVID-19 crisis, and a need to better understand resilience protective factors for their wellbeing in the post-pandemic period.

Resilience is a dynamic process of positive adaptation that occurs in response to adversity or stress and involves interaction between a range of protective factors and resources that are *personal* (e.g. optimism, cognitive flexibility, coping skills), *relational* (e.g. social support) and *environmental* (e.g. psychological support services) and help restore psychological wellbeing [18, 19]. The resilience process is relational and embedded in a person’s social ecology and involves the ability to navigate towards resilience resources which help sustain wellbeing, as well as the capacity to negotiate for those [20]. For nursing students, higher resilience has been associated with higher wellbeing over the course of a nursing degree [21]. Studies of nursing students during the pandemic, however, typically identified low resilience [22, 23], suggesting students were at risk for compromised wellbeing. A scoping review identified higher resilience in nursing students during the pandemic as predictive of higher psychological wellbeing, and that in the face of the pandemic, resilience was a valuable protective process against mental distress [24]. There is a need to better understand how resilience explored from a social ecological perspective influences nursing students’ wellbeing and mental distress.

Despite studies variously examining the mental distress, coping and wellbeing of nursing students during the COVID-19 pandemic, there is no prior mixed methods research providing comprehensive evidence on the impacts of the pandemic on students’ mental distress, coping, wellbeing and resilience, as well as the personal, environmental, and relational resilience resources they drew on to navigate pandemic challenges. Further, there is very limited research investigating the impact of the pandemic on the mental distress and wellbeing of international versus domestic nursing students [4, 25, 26]. International nursing students have reported lower depression, anxiety and stress than domestic students [26], but may have greater psychosocial support needs due to a lack of family and social support [4]. As university teaching and learning transitions into the post-pandemic era, nursing students who began or completed their degrees during the height of the COVID-19 crisis are now entering the nursing workforce or about to graduate as registered nurses. As such, we are yet to fully understand the implications of the pandemic for this emerging workforce. This study addresses these gaps in knowledge. The overall aim was to investigate the wellbeing, coping and resilience of undergraduate nursing students during the pandemic, and explore the resilience resources students used to deal with pandemic adversity.

Specific objectives were to:

1. Describe the demographics, mental distress, wellbeing, coping and resilience of nursing students, explore relationships between these variables, and investigate predictors of wellbeing and coping, including differences between domestic and international students (Quantitative Phase).
2. Explore how students experienced and managed adversity (Qualitative Phase).
3. Understand how students’ mental distress and wellbeing were influenced by the resilience resources they drew on to deal with adversity (Integration Phase).
4. Identify implications for nurses’ wellbeing as they enter the workforce in the post-pandemic era.

## Methods

### Research design

A convergent mixed methods design was used. This approach enables more comprehensive understanding of a phenomenon than one method alone [27]. The study is reported using The Mixed Methods Appraisal Tool [28].

### Study setting

Data were collected nationally between March-August 2022 at a public national Australian university with six campuses offering undergraduate nursing across the Eastern seaboard of Australia.

### Participants

Students enrolled in 1st, 2nd and 3rd years of undergraduate nursing degrees (Bachelor of Nursing, and Bachelor of Nursing/Bachelor of Business Administration double degree students) were eligible. The total eligible student population was *n* = 1958, with *n* = 496 (25.33%) of these being international students. Participants were primarily contacted through their course website with a flyer and embedded survey link posted on the Learning Management System. The online REDcap survey was also advertised in tutorials using a QR code. At the end of the survey, students were invited to be contacted for an interview.

### Ethics

Ethical approval was granted from the Australian Catholic University Human Research Ethics Committee (approval number 2021-311E). An information sheet was provided at the start of the online survey, outlining the study purpose and procedures. Participation was voluntary and all participants gave written informed consent to take part by selecting “Yes, I agree to participate”, at the beginning of the survey. For the interview phase of the study, audio-recorded verbal assent was obtained from participants prior to commencing the interview.

### Data collection

#### Survey

*Demographics* included questions on age, gender, marital status, country of birth, course, enrolment year, enrolment status (part vs. full time), student status (domestic vs. international), and employment status. See Table 1.

**Table 1:** Demographic information

Variable		*N* (Survey data)		*N* (Qualitative interviews)
Age	< 20	28		3
	20–29	93		12
	30–39	15		2
	40+	9		-
	Missing	30		1
	Mean (SD)	24.95 (7.41)		24.18 (5.34)
Gender	Male	18		3
	Female	154		14
	Other	2*		1^
	Missing	1		-
Marital Status	Single	83		8
	In a relationship	63		8
	Married / De facto	27		2
	Separated	2		-
Country of Birth	Australia (105), Cambodia (1), China (5), Colombia (1), Hong Kong (1), India (3), Iraq (1), Ireland (1), Kenya (1), Korea (1), Malaysia (1), Nepal (20), New Zealand (4), Philippines (9), Romania (1), South Korea (1), South Sudan (1), Sri Lanka (1), Thailand (2), UK (2), Vietnam (5), Missing (8).			Australia (13), Nepal (2), New Zealand (1), Philippines (2).
Enrolment year	First	32		6
	Second	96		6
	Third	47		3
Studying full or part time	Full-time	154		18
	Part-time	21		-
Degree	Bachelor of Nursing	164		16
	Bachelor of Nursing / Bachelor of Business Administration	11		2
Student Status (Domestic vs. International)	Domestic	133		15
	International	42		3
Employment Status	Full-time	14		1
	Part-time	81		13
	Casual	69		4
	Not employed	11		-

Note: * one participant identified as non-binary and one participant identified as a transgender man. ^ one participant identified as non-binary

*Wellbeing* was measured using the COMPAS-W Wellbeing Scale, a composite measure of wellbeing or mental health [29]. This measure sums scores on 26 items rated on a Likert scale from 1 (strongly disagree) to 5 (strongly agree), with higher scores indicating higher wellbeing. Summed total scores range from 26 to 130 and are categorised as languishing (< 88), moderate (89–108) or flourishing (109+). Excellent reliability has been previously reported (α = 0.90; [29]) and good reliability was found in the current study (α = 0.86).

*Mental distress* was measured using the Depression Anxiety Stress Scales (DASS-21; [30]), completed on a 4-point Likert scale from 0 (did not apply to me at all) to 3 (applied to me very much, or most of the time), with subscales for each construct. Scores range from 0 to 21 and are multiplied by 2 to align with the DASS-42 scoring system. Higher scores indicate greater mental distress. For each subscale, scores are categorised by severity ranging from normal to extremely severe (See Table 2). Good reliability has been previously reported (Depression α = 0.82, Anxiety α = 0.79, Stress α = 0.86; [9]), and good to excellent reliability was found in the current study (Depression α = 0.92, Anxiety α = 0.89, Stress α = 0.89).

**Table 2:** Number and proportion of participants for DASS clinical cutoffs

Severity	Depression			Anxiety			Stress		
	*N*	%	Clinical cutoff	*N*	%	Clinical cutoff	*N*	%	Clinical cutoff
Normal	68	47.89	0–9	57	39.04	0–7	80	56.3	0–14
Mild	18	12.68	10–13	7	4.79	8–9	15	10.6	15 − 8
Moderate	31	21.83	14–20	26	17.81	10–14	17	12.0	19–25
Severe	12	8.45	21–27	20	13.70	15–19	17	12.0	26–33
Extremely severe	13	9.15	28+	36	24.66	20+	13	9.2	34+

*Coping style* was assessed with the 28 item Brief COPE [31], using a 4-point format, from 1 (“I haven’t been doing this at all”) to 4 (“I’ve been doing this a lot”). Items are categorised into three factors: (1) emotion focused coping (2) problem focused coping or (3) avoidance focused coping. Scores are calculated as the average of all items belonging to a specific factor. Higher scores indicate more use of that coping style. Emotion and problem focused coping are typically considered adaptive strategies, with avoidance coping considered maladaptive [31]. Questionable to good reliability has been previously reported for this measure study (emotion-focused α = 0.75, problem-focused α = 0.81, avoidant-focused α = 0.68; [32]), however good reliability was found in this study (emotion-focused α = 0.81, problem-focused α = 0.85, avoidant-focused α = 0.79).

*Resilience* was assessed with the Adult Resilience Measure Revised (ARM – R), which is based on a social ecological perspective of resilience that considers both individual characteristics and social relationships [33]. Seventeen items are rated on a 5-point Likert scale from 1 (not at all) to 5 (a lot). Scores range from 17 to 85, and can be categorised as low, moderate or high resilience, based on the sample median (34). Good reliability has been previously reported (α = 0.87; [34]) and excellent reliability was found in this study (α = 0.94).

#### Interviews

Individual or focus group semi-structured interviews [27] were offered depending on student availability. These were conducted by an experienced qualitative researcher (KF) and audio-recorded online via Zoom. Interviews/focus groups ranged from 30:30–55 min, with an average of 43.7 min. The open-ended interview questions are consistent with resilience theory and were developed by a committee of expert resilience researchers. Questions, with prompts as relevant, explored students’ experiences during the pandemic (e.g. ‘What has been your experience of learning during the pandemic?), challenges they faced (e.g. ‘What particular challenges have you faced?’), and how they dealt with those challenges (‘Please tell us about the ways in which you have responded to these challenges’).

### Data analysis

#### Quantitative

Data were processed and analysed with R Programming version 4.4.1 [35]. Interaction effects were interpreted using a simple slopes analysis from the ‘ggeffects’ package [36]. Means and standard deviations were reported for continuous outcome measures, and frequencies and percentages for categorical measures (see Table 3). Due to non-normal data, Spearman’s rank correlations were conducted to explore relationships between wellbeing, resilience, coping, mental distress, and age. A combination of independent samples t-tests (for normally distributed variables) and Mann-Whitney U Tests (for non-normally distributed variables) examined differences in wellbeing, resilience, and mental distress between domestic and international students. One-way ANOVAs were used to examine differences in wellbeing, resilience, coping, and mental distress among first, second, and third-year students. To determine significant predictors of wellbeing and coping (emotion-focused, problem-focused and avoidance-focused), four backward stepwise regressions were conducted. All initial models included predictors of depression, anxiety, stress, emotion-focused coping, problem-focused coping, avoidance-focused coping, wellbeing, age, enrolment year, student status, resilience and an interaction between resilience and student status. To make interactions more interpretable, resilience was centred around the mean. Variables were removed one at a time based on their contribution to the model fit, as measured by the Akaike Information Criterion (AIC; [37]). This approach may retain non-significant predictors if they contribute to the overall model fit and complexity. Multicollinearity was assessed via the Variance Inflation Factor, which was acceptable (< 5) for all predictors in all models. For all statistical tests, significance was accepted at *p* <.05.

**Table 3:** Descriptive statistics for outcome measures

Measure	Mean (SD)		Minimum		Maximum	
	Whole sample	Qualitative sample	Whole sample	Qualitative sample	Whole sample	Qualitative sample
DASS – Depression	11.55 (10.44)	12.47 (13.2)	0	0	42	38
DASS - Anxiety	12.59 (10.53)	14.47 (12.1)	0	0	40	40
DASS - Stress	15.58 (10.50)	18.5 (12.1)	0	0	40	40
Wellbeing	90.54 (11.79)	88.2 (16.1)	45	45	116	106
Resilience	69.64 (11.53)	66.94 (15.7)	17	17	85	85
Emotion Coping	2.21 (0.57)	2.32 (0.61)	1	1	3.82	3.75
Problem Coping	2.46 (0.69)	2.49 (0.70)	1	1	4	3.36
Avoidance Coping	1.79 (0.56)	1.73 (0.57)	1	1	3.25	3.25

#### Qualitative

Audio-recorded interviews and focus groups with *n* = 18 participants were transcribed verbatim and de-identified. The range and depth of data generated was sufficient to provide ‘information power’ and to answer the research objective, i.e. it provided data sufficiency [38]. Qualitative data were analysed using thematic analysis. Analysis was undertaken according to Saldaña [39], where researchers (LA, KF, TH) initially read transcripts and independently identified descriptive codes in first level coding. These were then grouped into categories in the second stage of coding. Categories were developed into emergent themes and discussed and refined by the researchers until they reached consensus.

#### Mixed methods

To understand how mental distress and wellbeing were influenced by the resilience resources used to cope with challenges, mixed methods analysis was conducted to integrate quantitative and qualitative data. This comprised data reduction, data display, data transformation, data consolidation, data comparison and data integration [40]. Both sets of data were reduced and displayed in a visual format and then transformed. The quantitative data (means and correlations for DASS & COMPAS-W) were transformed into narratives. Qualitative data were deductively content analysed for personal, relational, and environmental resources. The transformed data were synthesised and correlated, with comparisons across datasets, and then combined to create an integrated set of findings and meta-inferences (see Table 6).

## Results

### Participant demographics

The final survey sample consisted of 175 nursing students, indicating a response rate of 8% (out of potential population *n* = 1958). Students were aged between 18 and 55. For the qualitative interviews, the sample comprised 18 participants aged between 19 and 38. See Table 1.

### Wellbeing, mental distress, resilience, and coping descriptives

Descriptive statistics for wellbeing (COMPAS-W), emotional distress (DASS-21), coping style (Brief COPE) and resilience (ARM-R) are presented in Table 3. Most students reported moderate to flourishing wellbeing (59%), however 41% reflected languishing. For mental distress, participants in each clinical cutoff category are shown in Table 2. Over a third of students (39%) reported moderate to severe depression, over half (56%) reported moderate to severe anxiety, and a third (33%) reported moderate to severe stress. Most of the sample (67%) had moderate resilience scores, with 17% reporting low, and 16% reporting high, resilience. Coping strategies were interpreted by examining the mean for each coping style (emotion focused, problem focused, and avoidance focused). Based on the mean, problem-focused coping was the most utilised strategy, followed by emotion-focused coping, and avoidant coping.

### Correlations and preliminary analyses

Correlations between all continuous variables are shown in Table 4. In summary, higher wellbeing was significantly associated with lower mental distress, less emotion-focused coping, less avoidance coping, and higher problem-focused coping and resilience. All constructs of mental distress were significantly positively associated with each other, and with emotion-focused and avoidance-focused coping. All constructs of mental distress were significantly negatively associated with resilience and age. Higher levels of depression, anxiety and stress were associated with more use of emotion and avoidant focused coping, lower resilience, and being younger.

**Table 4:** Correlations between variables

Variable	1	2	3	4	5	6	7	8	9
1. Wellbeing	-								
2. DASS -Depression	− 0.553**	-							
3. DASS – Anxiety	− 0.394**	0.727**	-						
4. DASS – Stress	− 0.469**	0.771**	834**	-					
5. Emotion Coping	− 0.228*	0.512**	0.373**	0.486**	-				
6. Problem Coping	0.185*	0.076	0.099	0.116	0.581**	-			
7. Avoidance Coping	− 0.387**	. 646**	0.421**	0.511**	0.614**	0.276*	-		
8. Resilience	0.442**	− 0.347**	− 0.262*	− 0.281**	− 0.053	0.250*	− 0.098	-	
9. Age	0.106	− 0.236*	− 0.259*	− 0.227*	− 0.023	0.203*	− 0.152	0.067	-

**Significant at *p* <.001, *significant at *p* <.05

Full details of Mann-Whitney U and independent t-tests are reported in Supplementary Tables 1 and 2. There were no significant differences in outcomes based on enrolment year (*p* >.05). A Mann-Whitney U test revealed statistically significant differences in stress levels between domestic and international students (U = 330.5, *p* =.010). Domestic students reported significantly higher levels of stress (*Mdn* = 14) compared to international students (*Mdn* = 10).

### Linear regressions

A full summary of the regressions is provided in Table 5.

#### Wellbeing

The final model included depression, emotion-focused coping, problem-focused coping, resilience, student status and an interaction between resilience and student status. Lower depression (*p* <.001) and increased problem-focused coping (*p* =.005) significantly predicted greater wellbeing. There were no significant main effects of resilience, emotion-focused coping or student status (*p* >.05), but they were retained due to their overall contribution to the model fit. Additionally, resilience moderated the relationship between student status and wellbeing, where, as resilience increased, domestic students had a greater increase in wellbeing compared to international students. At high levels of resilience, domestic students had significantly higher wellbeing than international students (*p* =.011).

#### Emotion-focused coping

The final model included depression, anxiety, stress, avoidance-focused coping, problem-focused coping, enrolment year and student status. Higher stress (*p* =.020), more avoidance-focused coping (*p* <.001) and problem-focused coping (*p* <.001) were significantly associated with increased emotion-focused coping. Second year students used significantly less emotion-focused coping compared to first-year students. There was no significant main effect of depression, anxiety, or student status on emotion-focused coping (*p* >.05), but they were retained due to their contribution to the overall model fit.

#### Problem-focused coping

The final model included anxiety, emotion-focused coping, wellbeing, student status, resilience, age and an interaction between student status and resilience. Higher wellbeing (*p* <.001), emotion-focused coping (*p* <.001), resilience (*p* =.006) and being older (*p* =.023) were significantly associated with increased problem-focused coping. The main effects of anxiety and student status, and the interaction between student status and resilience were not significant (*p* <.05) but were retained due to their contribution to the overall model fit.

#### Avoidance-focused coping

The final model included depression, anxiety, emotion-focused coping and age as predictors. Higher depression (*p* <.001) and more emotion focused coping (*p* <.001) were significantly associated with increased avoidance-focused coping. There was no significant main effect of anxiety or age on avoidance-focused coping, however they were retained in the final model due to its contribution to the overall model fit.

**Table 5:** Backwards step regressions

Outcome variables																			
Predictor variables	Wellbeing				Emotion-focused coping					Problem-focused coping						Avoidance-focused coping			
	β coefficient Initial model	β coefficient Final model	*p*-value		β coefficient Initial model	β coefficient Final model	*p*-value				β coefficient Initial model	β coefficient Final model	*p*-value				β coefficient Initial model	β coefficient Final model	*p*-value
Wellbeing	-	-			-0.085						0.270	0.338	< 0.001				-0.024		
Depression	-0.520	-0.562	< 0.001		0.106	0.177	0.128				-0.128						0.522	0.547	< 0.001
Anxiety	0.006				-0.207	-0.188	0.131				0.264	0.130	0.158				-0.148	-0.186	0.083
Stress	-0.071				0.296	0.304	0.020				-0.117						0.063		
Emotion focused coping	-0.106	-0.154	0.126		-	-					0.733	0.667	< 0.001				0.451	0.398	< 0.001
Avoidance focused coping	-0.021				0.324	0.339	< 0.001				-0.25						-		
Problem focused coping	0.243	0.259	0.005		0.534	0.492	< 0.001				-						-0.025		
Age	0.025				-0.044						0.166	0.174	0.023				-0.109	-0.112	0.134
Enrolment year: Second	0.105				-0.185	-0.208	0.014				0.070						0.135		
Enrolment year: Third	0.048				-0.139	-0.160	0.060				0.082						0.038		
Student status: Domestic	0.124	0.109	0.113		-0.068						-0.082	-0.109	0.143				-0.087		
Resilience	-0.060	-0.044	0.722		-0.116						0.324	0.361	0.006				0.008		
Resilience: Student Status (Domestic)	0.318	0.296	0.011		0.087						-0.266	-0.249	0.051				0.046		
Adjusted R^2^	0.54	0.56			0.62	0.63					0.49	0.50					0.50	0.48	

### Qualitative findings

Two themes were derived from analysis: Grappling with life and learning challenges; and Learning to cope with adversity: unexpected benefits. For participant quotes, interview or focus group numbers are used (e.g. IV 1 or FG 1), and I/N for international or Dom for domestic students.

#### Grappling with life and learning challenges

Students faced several challenges including the transition to online learning. This was compounded by being in crowded personal living situations not conducive to studying from home full-time. Students found the online learning environment and lack of relationship with tutors negatively impacted their learning and motivation and affected their mental health. Several students perceived that due to the lack of contact, they did not learn and felt they had done their degree by themselves:

…during the pandemic I didn’t learn anything. I was like really, really disappointed in myself. It really took a toll on my mental health. “Oh my God, you’re getting worse and your friends are – like they’re achieving things”. (FG 1, I/N).

Some also noted they did not receive email responses from academics, or were not comfortable seeking help as it was difficult to articulate their request in an email, and when they did seek help, they were often referred to yet another online service:

A lot of these things are now done online and I found that to be a bit difficult, especially when you need help with something because you’re struggling with assessments…and it’s just another thing to add to the pile; it’s like working out how the services work, if you reach out to someone, they might just give you a list of guidelines through an email and it’s like ‘Well OK, I need to meet with someone to discuss this, and now I’m being given more online information (IV 3, Dom).

This concern was exacerbated for students with existing conditions which included anxiety and panic, and autism spectrum disorder, who found it difficult to adjust to full online learning and not being able to meet with someone to assist them. Students were also dissatisfied with the level of peer engagement in online classes, including peers not putting their camera on. Several found when they were sent to Zoom breakout rooms “there would just be like no one talking, complete black screens…that made me disengage” (IV 3, Dom).

These challenges intersected with students’ personal life, which included anxiety about catching COVID, challenges juggling study with paid employment, and severe financial hardship “…I lost my job…and ended up moving back (home)” (IV 3, Dom). While domestic students were often able to lean on family both emotionally and financially, some international students struggled:

…there were times where I was so bad in a situation where I can say like, I couldn’t pay my rent because of the pandemic. It gave me so much stress and I couldn’t concentrate…I’d just try to console myself saying – convince myself, so I’m like “hey, just go on” and then yes, it’s alright (FG 2, I/N).

Due to international border closures, international students were unable to return home even for funerals:

…I lost a lot of family members and relatives to COVID and it was hard…one second they are there, now they don’t even exist (FG 1, I/N).

Several students identified unresolved and ongoing effects from lockdowns, including loss of relationships and social networks.

#### Learning to cope with adversity: unexpected benefits

To address these challenges, students needed to be highly self-motivated and organised and to self-direct their learning. They used a range of coping strategies to manage their online and practical learning including having a routine and improving their technology skills. They noted the importance of adapting to the ever-changing environment and learning to be flexible and accepting and make the best of change and the situation, reframe their expectations, and understand it would not go on forever. Being realistic was also important:

near enough is good enough…the whole world is falling apart…it’s never going to be perfect (FG 5, Dom).

Despite their challenges, stories of resilience and ingenuity in problem-solving were common. As one student explained, it was important to look after themselves and address issues, so they didn’t linger and cause more stress:

If I had a problem or something that I’m stressed about, I just do it right away, so that I would finish it; I would face the problem and not prolong my suffering. And…I gave time to myself to do what I love (FG 1, I/N).

International students found alternative ways to get their financial needs met, including seeking work that wasn’t ‘person facing’ because they were not covered by Medicare if they became ill with COVID, and to have their learning skills met through actions like befriending a next-door neighbour who was a doctor and offered to teach them practical skills:

I went to his house regularly and he taught me how to do the vitals (FG 1, I/N).

While many students identified struggles with managing new technology such as *Zoom*, *YouTube* and other online platforms, they also acknowledged these were important new skills to acquire:

I didn’t even know *Zoom* existed before COVID…so getting to know how to use those systems (*Zoom*, *MS Teams*) and troubleshoot it…has been helpful (FG 5, Dom).

Others identified being pushed into online learning meant they had to become familiar with resources such as the virtual librarian, citation management programs, and online study tools (e.g., *Studiosity*, *Grammarly*): “…those (online services) have been really good, as accessing support virtually” (IV 4, Dom). Several students noted they now liked online learning better.

Lockdowns also enabled some to rest and regain energy, and others developed gratitude, resilience, enhanced personal growth, and spirituality. One student spoke of how her schooling and upbringing had helped her deal with challenges:

we’re like taught to be resilient, and so like whatever happens, you just have to go through it … (FG 1, I/N).

Students became better at time management and self-care, including taking care of their mental health and trying not to ruminate, and eating healthily, exercising, doing yoga, listening to music and spending time with family. For one student, clinical debrief sessions with a clinical educator led to referral to counselling services. Others sought counselling directly and used strategies such as cognitive behavioural therapy and body scans. They found these resources helped to mitigate the stress of lockdowns and online learning.

### Integration of findings and meta-inferences

To address the data integration objective, after the individual datasets were analysed a mixed methods analysis of both datasets was conducted. See Table 6. The findings of both datasets were initially displayed using tables. Quantitative data were transformed into narratives, and qualitative data were content analysed for personal, relational, and environmental resilience resources. Both datasets were reduced and compared for similarities and differences.

**Table 6:** Data display table of integration findings and meta-inferences

	Quantitative data	Qualitative data
Data display	• Overall severe depressive and stress symptoms, and extremely severe anxiety symptoms in the overall and qualitative samples. Extremely severe stress in the qualitative sample.	• Students experienced low mood, stress and anxiety related to catching COVID, social and peer isolation, online learning & clinical skills challenges, and difficulty accessing learning support.
	• Flourishing levels of wellbeing in overall sample, and moderate levels in qualitative sample.	• Students supported their wellbeing through increased self-care, problem-solving, learning new skills, and seeking technical and emotional support.
	• Moderate levels of resilience in overall and qualitative samples	• Students dealt with adversity through being proactive and drawing on or developing a range of coping strategies to manage challenges.
	• Resilience moderated the relationship between student status and wellbeing	• In adversity students learnt to manage their learning better, prioritise their physical and mental wellbeing, and strengthen their resilience and personal growth.
	• Higher depression predicted lower wellbeing	
Data reduction and transformation	• Despite significant mental distress, students reported moderate to flourishing wellbeing and moderate resilience.	• Students faced substantial challenges during COVID-19 including mental distress and challenges to their learning.
	• When resilience levels were high, international students experienced lower wellbeing than domestic students.	• Students drew on a range of personal, environmental and relational resources to cope with these challenges to their wellbeing and learning.
		• Without access to the same supports as domestic students (e.g. family support), international students had to rely more on personal resources such as problem solving.
Data comparison and consolidation	• COVID-19 led to substantial student stress and adjustment due to social isolation and rapid changes to learning and acquisition of clinical skills. • In the face of this adversity, students had high mental distress but also moderate levels of resilience and wellbeing. • By drawing on a range of personal, environmental and relational resilience resources, students were able to navigate the adversity of COVID-19. • Personal resources involved gratitude, self-awareness, cognitive self-regulation, cognitive flexibility, cognitive reframing, physical and psychological self-care, proactive problem solving, time management, active coping, stress management, and having a growth mindset. • Environmental resources involved access to educational, technical, financial, and clinical skills support, and counselling and employment opportunities. • Relational resources involved supportive relationships with family, peers and friends, educators, and counsellors.	
Data integration and meta-inferences	• Faced with the unique challenges of a global pandemic, most nursing students maintained their wellbeing and resilience, even in the presence of high distress. • Access to, and engagement with, personal, environmental and relational resilience resources served as protective factors for student wellbeing in this period of extraordinary challenge.	

Integration resulted in two main meta-inferences, or overall conclusions [27]. Faced with the unique challenges of a global pandemic, most nursing students maintained their wellbeing and resilience, even in the presence of high distress. Access to and engagement with personal, environmental and relational resilience resources served as protective factors for wellbeing during this extraordinary period.

## Discussion

This study investigated the experiences and outcomes of nursing students in respect to mental health and wellbeing, coping, and resilience during the pandemic. There were several novel findings that add to and extend existing knowledge, with implications for the post-pandemic era.

### Mental distress, wellbeing, coping and resilience

Students had high levels of distress (DASS-21), with over a third reporting moderate to severe depression, over half with moderate to severe anxiety, and a third with moderate to severe stress. These findings are generally consistent with other Australian studies during the pandemic using the DASS-21 e.g. [9, 26] but are markedly higher than some international studies e.g. [41]. Overall, higher distress was associated with greater use of emotion-focused and avoidant coping, lower resilience, and being younger. This study is the first to report this association between mental distress and coping in nursing students during the pandemic. Domestic students reported significantly more stress than international students, which is consistent with the limited prior literature, where international students reported lower stress scores on the DASS-21 [26]. It is possible, as Wynter et al. [26] suggest, that international students may have felt less stressed being in Australia during the pandemic due to the relatively lower prevalence of COVID infections compared to many other countries.

At the same time however, over half of students reported flourishing levels of wellbeing. A previous study with nurses also found flourishing wellbeing co-occurring with mental distress [18], although the current study is the first to report this with student nurses. These findings can be understood through the lens of the dual continua model of mental health, where mental health and wellbeing, and mental distress and illness, are considered related but distinct phenomena existing on separate continua. The presence of mental distress does not necessarily mean a lack of wellbeing [42]. Flourishing includes self-acceptance, supportive relationships, setting goals, personal growth, and a balanced life [43]. Our findings indicated that while students were distressed in the extraordinary circumstances of the pandemic, many had these characteristics and drew on these resilience resources which served as important protective factors for their wellbeing.

Findings also included a positive association between wellbeing and resilience, which is consistent with prior research. Reviews of nursing student literature have reported resilience to be positively associated with wellbeing [21, 44] and Smith et al., [24] found higher resilience during the pandemic predicted higher psychological wellbeing. Regression models in the current study showed wellbeing was predicted by lower depression and increased problem-focused coping. Our study is the first to report problem-focused coping as a predictor of wellbeing in nursing students during the pandemic. Problem-focused coping, which involves proactively addressing a problem to manage stress [45] promotes wellbeing, as confronting stressors directly can facilitate their removal or reduction [46]. In respect to coping and resilience, students overall reported healthy coping, with problem-focused the most utilised coping strategy and avoidance-focused the least. Problem-focused coping was associated with higher resilience, wellbeing, and being older. Usher et al., [9] also reported problem-focused coping to be the most utilised strategy, although they did not correlate their results with wellbeing and resilience.

### Resilience-promoting resources and mental distress and wellbeing

In this study, the quantitative findings indicate that resilience was a valuable protective process against students’ mental distress, and for their wellbeing. Two-thirds of students reported moderate resilience, in contrast to most studies reporting low resilience of nursing students during the pandemic [22, 23]. Usher et al. [9] however, also found moderate resilience in their Australian study. This finding is complemented by the mixed methods integration, where the qualitative findings indicated a range of relational, personal and environmental resilience resources were used by students. Relational support was a strong resilience-promoting factor, which is consistent with Henshall et al.’s [11] findings and is a robust factor well-recognised in the wider resilience literature [20]. Personal resources used by students included cognitive flexibility, problem-solving, and having a growth mindset, where they displayed a belief in their ability to continue learning and growing [47]. These are important resilience-promoting factors which can be learned and developed through wellbeing and resilience education [48]. In this study, environmental resources included having access to educational, technical, financial and psychological supports. To enable restoration of their wellbeing in adversity, these crucial external resources [20] need to be made available and accessible to students.

### Implications for graduate nurse wellbeing in the post-pandemic era

The findings have several key implications for nursing students. To strengthen students’ coping and wellbeing prior to their entry to the workforce, and to support their transition into practice as newly graduated registered nurses, it is recommended that universities provide wellbeing and self-care education during undergraduate nursing education that includes strategies to promote adaptive coping. The findings on mental health, wellbeing and resilience also have implications for the post-pandemic era and supporting current and future nursing graduates now entering the workforce, the majority of who in this study were young people up to 29 years. There are recognised longitudinal mental health and wellbeing impacts of the pandemic for the community, and mental distress overall remains higher than pre-COVID levels [49]. Given the specific risk of ongoing mental distress for new graduates as they acculturate to the practice environment, it is recommended tailored wellbeing education which includes self-care strategies and adaptive coping skills are provided during transition, and psychological and peer wellbeing support is offered as needed. To promote graduates’ resilience during their transition to practice, it is recommended that health services provide graduate nurses with resilience education which includes specific resilience resources they can draw on such as those identified in the current study, and practice-related professional development.

### Limitations

This study was conducted with students at one university in one country context, and findings may not be transferable to other settings. The survey response rate was low, although it was consistent with some other nursing student studies [50]. Due to the survey being primarily accessible through the online Learning site, it is unclear how many students accessed the survey link. The results may not be representative of the larger student population and may not be generalisable. Participants self-selected into the study and their experiences may be different to those who did not participate. The cross-sectional survey design provided data at one time point only, and causal inferences cannot be made.

## Conclusion

Health services can help strengthen new graduate nurses’ wellbeing and retention through enhancing their workplace sense of belonging, where they feel valued and appreciated by colleagues and managers, and implementing strategies to build supportive relationships and connections within teams. In respect to research and to support retention, longitudinal follow-up of graduate nurses entering the workforce is needed to ascertain how they cope over time, and to inform how health services can best meet graduates’ ongoing mental health and wellbeing support needs.

## Electronic supplementary material

Below is the link to the electronic supplementary material.

Supplementary Material 1

## Data Availability

The datasets generated and analysed are not publicly available due to privacy and ethical restrictions, but are available from the corresponding author on reasonable request.
