# Supplementary material for: A mixed methods study of wellbeing and resilience of undergraduate nursing students: implications for the post-pandemic era
Source: BMC Nurs. 2025 Apr 11;24:409. doi: 10.1186/s12912-025-03066-0 (PMC11987187; doi:10.1186/s12912-025-03066-0)
Supplement: Supplementary file 1 — Supplementary Material 1 [file 12912_2025_3066_MOESM1_ESM.docx]

| **Supplementary Table 1. Impact of student status on outcome variables.** | | | | | | |
| --- | --- | --- | --- | --- | --- | --- |
| **Outcome** | **Test Type** | **Statistic** | **df** | ***p*** | **Mean (*SD*)**  **Domestic Students** | **Mean (*SD*)**  **International Students** |
| Wellbeing | Mann-Whitney | *W* = 2772 | - | .633 | 90.52 (11.27) | 90.60 (13.48) |
| Depression | Mann-Whitney | *W* = 1446.5 | - | .123 | 12.00 (10.18) | 10.00 (11.34) |
| Anxiety | Mann-Whitney | *W* =1398.5 | - | .076 | 13.27 (10.33) | 10.25 (11.03) |
| **Stress** | **Mann-Whitney** | ***W* = 1233.5** | **-** | **.010** | **16.67 (10.23)** | **11.81 (10.70)** |
| Emotion coping | T-test | *t* = 0.781 | 48.05 | .439 | 2.19 (0.54) | 2.28 (0.63) |
| Avoidance coping | Mann-Whitney | *W* = 1462.5 | - | .959 | 1.79 (0.55) | 1.80 (0.59) |
| Problem coping | T-test | *t*= 0.615 | 48.88 | .545 | 2.44 (0.67) | 2.53 (0.75) |
| Resilience | Mann-Whitney | *W* =2097 | - | .769 | 69.90 (10.33) | 68.77 (14.97) |

| **Supplementary Table 2. Impact of enrolment year on outcomes variables.** | | | | | | | |
| --- | --- | --- | --- | --- | --- | --- | --- |
| **Outcome variable** | **Test Type** | **Statistic** | **df** | ***p*** | **Mean (*SD*)**  **First Year** | **Mean (*SD*)**  **Second Year** | **Mean (*SD*)**  **Third Year** |
| Wellbeing | Kruskal-Wallis | χ² = 5.02 | 2 | 0.08 | 94.19 (9.96) | 88.97(12.87) | 91.17 (10.14) |
| Depression | Kruskal-Wallis | χ² = 0.79 | 2 | 0.67 | 10.00 (10.18) | 12.11 (10.59) | 11.48 (10.48) |
| Anxiety | Kruskal-Wallis | χ² =0.03 | 2 | 0.98 | 12.08 (9.84) | 12.72 (10.54) | 12.67 (11.14) |
| Stress | Kruskal-Wallis | χ² =0.32 | 2 | 0.85 | 16.40 (10.75) | 15.47 (10.08) | 15.29 (11.29) |
| Emotion coping | ANOVA | *F*= 1.04 | 2, 121 | 0.36 | 2.36 (0.61) | 2.16 (0.60) | 2.21 (0.46) |
| Avoidance coping | Kruskal-Wallis | χ² = 1.50 | 2 | 0.47 | 1.73 (0.59) | 1.87 (0.63) | 1.69 (0.38) |
| Problem coping | ANOVA | *F* = 1.37 | 2, 121 | 0.26 | 2.49 (0.76) | 2.37 (0.74) | 2.60 (0.54) |
| Resilience | Kruskal-Wallis | χ² = 0.20 | 2 | 0.91 | 70.31 (11.03) | 68.86 (12.94) | 70.76 (8.63) |
